# Rampant intraspecific variation of plastid genomes in *Gentiana* section *Chondrophyllae*

**DOI:** 10.1002/ece3.70239

**Published:** 2024-09-02

**Authors:** Shan‐Shan Sun, Zhi‐Yong Pan, Yu Fu, Shen‐Jue Wang, Peng‐Cheng Fu

**Affiliations:** ^1^ School of Life Science Luoyang Normal University Luoyang China

**Keywords:** *Gentiana*, intraspecific variation, plastome, section *Chondrophyllae* s.l

## Abstract

Exploring the level of intraspecific diversity in taxa experienced radiation is helpful to understanding speciation and biodiversity assembly. *Gentiana* section *Chondrophyllae* sensu lato encompasses more than 180 species and occupies more a half of species in the genus. In this study, we collected samples across the range of three species (*Gentiana aristata*, *G. crassuloides* and *G. haynaldii*) in section *Chondrophyllae* s.l., and recovered the intra‐species variation by comparing with closely related taxon. Using 25 newly sequenced plastid genomes together with previously published data, we compared structural differences, quantified the variations in plastome size, and measured nucleotide diversity in various regions. Our results showed that the plastome size variation in the three *Chondrophyllae* species ranged from 285 to 628 bp, and the size variation in LSC, IR and SSC ranged from 236 to 898 bp, 52 to 393 bp and 135 to 356 bp, respectively. Nucleotide diversity of plastome or any of the four regions was much higher than the control species. The average nucleotide diversity in plastomes of the three species ranged from 0.0010 to 0.0023 in protein coding genes, and from 0.0023 to 0.0061 in intergenic regions. More repeat sequence variations were detected within the three *Chondrophyllae* species than the control species. Various plastid sequence matrixes resulted in different backbone topology in two target species, showed uncertainty in phylogenetic relationship based inference. In conclusion, our results recovered that species of *G.* section *Chondrophyllae* s.l. has high intraspecific plastome variation, and provided insights into the radiation in this speciose lineage.

## Graphical abstract

Significant high level of intraspecific plastome size variation and genetic diversity are detected in three annual gentians of *Gentiana* section *Chondrophyllae* s.l.

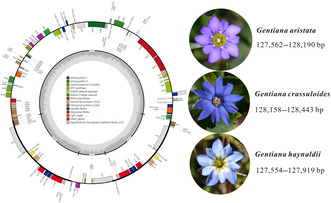

## 1 INTRODUCTION

The increasing availability of plastid genomes (plastome) represents an excellent opportunity to explore evolutionary processes in plants (Sibbald & Archibald, 2020; Twyford & Ness, 2017). For example, plastid phylogenomics led to a better understanding of evolutionary patterns in taxa experienced radiation or rampant hybridization, as the case of evolutionary radiations in *Saussurea* (Zhang et al., 2021). Plastome structure is usually conservative in land plants, composing of two inverted repeat (IR) regions that are separated by the large single copy (LSC) region and the small single copy (SSC) region (Jansen & Ruhlman, 2012; Mower & Vickrey, 2018). Comparative analysis among closely related taxa have detected a number of plastome microstructural changes including expansion/contraction or loss of the IR (Lee et al., 2021; Wang et al., 2022) and gene loss (Mower et al., 2021; Wang et al., 2023). Linking the changes with diversification can offer clues to the mechanisms driving their evolution (Fu et al., 2021; Wang et al., 2023; Wicke et al., 2016). Besides phylogeny and molecular evolution, rapid increasing data also offer great benefits to explore intraspecific plastome variation in some diverse groups experienced rapid evolution. Accelerated plastid genome evolution was speculated to contribute to the early stages of speciation (Barnard‐Kubow et al., 2014). Intraspecific variation in plastome sequence and structure has been reported for a few groups of angiosperms, for example the variation in IR extent and boundaries of junction sites in *Medicago minima* (Choi et al., 2020), length variation in coding region in *Medicago truncatula* (Gurdon & Maliga, 2014), and gene degradation at individual and population levels in *Cymbidium* (Kim & Chase, 2017).

*Gentiana* is a typical alpine genus, with the Qinghai‐Tibet Plateau (QTP) acting as the main center of diversity and the primary source area for dispersal to mountainous areas of the world (Favre et al., 2016; Ho & Liu, 2001). Although *Gentiana* is distributed in mountain systems around the world, in fact, section *Chondrophyllae* Bunge sensu lato (s.l.) is the only section globally distributed, whereas another 11 sections of *Gentiana* are endemic to one or two continents (Ho & Liu, 2001). Section *Chondrophyllae* s.l composes about 182 species, occupying 51.7% of all *Gentiana* species. Section *Chondrophyllae* s.l. is a well‐supported monophyletic group with a long branch in phylogenetic tree (Favre et al., 2020; Fu et al., 2021), hitting rapid evolution in this group (Yuan & Küpfer, 1997). In subtribe Gentianinae, section *Chondrophyllae* s.l. has experienced the most notable plastome variations including plastome size decreases, gene loss, IR contraction and SSC reduction (Fu et al., 2022), and were supposed to be correlated with the rapid evolution in this section (Fu et al., 2021). In addition, previous studies showed obvious cyto‐nuclear conflict in section *Chondrophyllae* s.l. (Chen et al., 2021; Fu et al., 2022). Although hybridization is widely accepted to explain cyto‐nuclear conflict, conflicting phylogenetic signals in the plastome which had been observed in various lineages (Walker et al., 2019) are not yet assessed in *Gentiana*. Since the plastome is rather dynamic in section *Chondrophyllae* s.l., we wonder what's the level of plastome variation in this lineage, and if the variation has impact on phylogenetic reconstruction.

In this study, we focus on three species, *Gentiana aristata* Maxim., *Gentiana crassuloides* Bureau & Franch. and *Gentiana haynaldii* Kanitz, which came from three series in section *Chondrophyllae* s.l. By sequencing samples across the range of the three species and comparing with species from the most closely related lineage of section *Chondrophyllae* s.l., we aim to recover the level of intraspecific plastome diversity and microstructural changes in section *Chondrophyllae* s.l., and furtherly assess its impact on reconstructing phylogenetic relationship.

## 2 MATERIALS AND METHODS

### 2.1 Studied species and sampling

The three target species, *Gentiana aristata*, *G. crassuloides* and *G. haynaldii*, belong to series *Humiles* Marquand, *Orbiculatae* Marquand and *Dolichocarpa* T.N.Ho, respectively (Favre et al., 2020; Ho & Liu, 2001). The three species are annual herbs, and endemic to the QTP. We sampled individuals from different localities to cover the entire range of each species. Because the three species are minute annuals, a whole single plant was collected in the wild for each individual, and conserved in silica gel prior to extraction. In total, individuals from 18, 15 and 10 localities were collected for *G. aristata*, *G. crassuloides* and *G. haynaldii*, respectively (Table 1). No permission to sample was needed because the target species were either in the List of National Key Protected Wild Plants China or sampled from nationally protected regions. *Gentiana crassicaulis* Duthie ex Burk. from section *Cruciata* Gaudin which is the sister group of section *Chondrophyllae* s.l. (Favre et al., 2020; Fu et al., 2021) was served as the control. Species were identified by Dr. Peng‐Cheng Fu, and voucher specimens were deposited either in the herbarium of Luoyang Normal University (no acronym at present).

**TABLE 1:** Information of newly sequenced plastid genomes in three species from *Gentiana* section *Chondrophyllae* s.l. *Gentiana crassicaulis* whose voucher no. was Genbank accession number, was served as the control.

Species	Voucher no.	Latitude	Longitude	Altitude (m)	GenBank no.	LSC	IR	SSC	Total
*G. aristata*	fu2016001	35°35′	102°42′	3094	PQ154566	73,779	22,336	9394	127,845
	fu2016016	34°42′	102°29′	3281	PQ154567	74,126	22,333	9398	128,190
	fu2017008	35°52′	99°58′	3360	PQ154561	73,773	22,347	9386	127,853
	fu2017031	33°07′	97°27′	4119	PQ154553	73,919	22,506	9206	128,137
	fu2017055	32°46′	97°12′	4013	PQ154557	74,247	22,404	9128	128,183
	fu2017112	31°04′	96°56′	4626	PQ154560	73,890	22,506	9208	128,110
	fu2017162	31°44′	100°43′	4022	PQ154558	73,731	22,335	9384	127,785
	fu2017195	32°09′	102°30′	3516	PQ154554	73,825	22,327	9407	127,886
	fu2017206	33°12′	101°28′	3717	PQ154552	73,827	22,327	9407	127,888
	fu2017240	33°27′	100°59′	3651	PQ154562	73,737	22,329	9388	127,783
	fu2017250	33°39′	99°46′	4152	PQ154563	73,725	22,217	9403	127,562
	fu2017276	34°18′	100°16′	3985	PQ154559	73,826	22,327	9407	127,887
	fu2017352	36°19′	101°29′	3600	PQ154569	73,985	22,113	9390	127,601
	fu2018016	34°27′	102°18	3603	PQ154564	74,075	22,317	9416	128,125
	fu2019139	37°07′	102°27′	2978	PQ154555	73,706	22,347	9421	127,821
	fu2019557	37°47′	101°19′	3640	PQ154568	73,706	22,347	9421	127,821
	fu2019677	38°00′	100°21′	3209	PQ154565	73,706	22,347	9443	127,843
	fu2019688	37°35′	100°45′	3439	PQ154556	73,705	22,347	9399	127,798
*G. crassuloides*	fu2016024	33°12′	102°24′	3545	PQ154539	72,961	22,525	10,232	128,243*
	fu2016094	29°51′	102°01′	3442	PQ154532	73,206	22,373	10,432	128,384*
	fu2017054	32°46′	97°12′	4013	PQ154534	72,985	22,562	10,310	128,419*
	fu2017086	31°08′	96°29′	4187	PQ154537	72,982	22,562	10,310	128,416*
	fu2017155	31°42′	99°37′	3752	PQ154536	73,068	22,375	10,479	128,297*
	fu2017163	31°44′	100°43′	4022	PQ154540	72,961	22,662	10,138	128,423*
	fu2017181	31°53′	102°38′	3343	PQ154528	72,308	22,373	10,468	128,422*
	fu2017194	32°09′	102°30′	3516	PQ154533	73,116	22,368	10,463	128,315*
	fu2017211	33°12′	101°28′	3717	PQ154531	72,898	22,383	10,494	128,158*
	fu2017241	33°27′	100°59′	3926	PQ154529	72,954	22,662	10,138	128,416*
	fu2017252	33°39′	99°46′	4152	–	72,954	22,662	10,138	128,416*
	fu2018053	28°20′	99°04′	4326	PQ154538	73,059	22,507	10,370	128,443*
	fu2018117	28°48′	97°42′	4094	PQ154541	73,071	22,502	10,297	128,372*
	fu2019003	34°00′	107°46′	3500	PQ154535	73,052	22,510	10,328	128,400*
	fu2022005	–	–	3600	PQ154530	72,958	22,547	10,346	128,398*
*G. haynaldii*	fu2016063	31°59′	99°05′	4021	PQ154542	73,532	22,124	10,117	127,897*
	fu2016075	30°59′	101°08′	3548	PQ154543	73,296	22,079	10,100	127,554*
	fu2017057	32°46′	97°12′	4013	PQ154551	73,531	22,124	10,117	127,896*
	fu2017067	32°35′	96°32′	4133	PQ154545	73,523	22,124	10,117	127,888*
	fu2017141	31°19′	98°04′	4160	PQ154549	73,531	22,124	10,117	127,896*
	fu2017154	31°42′	99°37′	3752	PQ154546	73,512	22,124	10,117	127,877*
	fu2017180	31°53′	102°38′	3343	PQ154544	73,521	22,131	10,136	127,919*
	fu2018034	27°48′	99°38′	3400	PQ154547	73,307	22,094	10,235	127,730*
	fu2020013	30°05′	91°16′	4268	PQ154548	73,524	22,124	10,117	127,889*
	fu2020024	29°42′	92°07′	4552	PQ154550	73,524	22,124	10,117	127,889*
*G. crassicaulis*		–	–	–	KY595457	81,143	25,272	17,070	148,757
		–	–	–	KY595458	81,143	25,272	17,070	148,757
		–	–	–	KY595459	81,174	25,272	17,070	148,788
		–	–	–	KY595460	81,162	25,272	17,071	148,777
		–	–	–	KY595461	81,164	25,272	17,070	148,778
		–	–	–	KY595462	81,141	25,272	17,070	148,755
		–	–	–	KY595463	81,140	25,272	17,070	148,754
		–	–	–	KY606171	81,110	25,272	17,070	148,724
		–	–	–	KJ676538	81,164	25,271	17,070	148,776

*Note*: Columns LSC, IR and SSC report the length (bp) of the large single‐copy, inverted repeat and small single‐copy regions, respectively. Newly sequenced plastid genomes were indicated with asterisks (*) behind the total length.

### 2.2 Sequencing, assembly and annotation

Total genomic DNA was isolated from dried leaves to perform genome‐skimming sequencing. A 500‐bp DNA Illumina sequencing library was constructed using about 4.0 ng of sonicated DNA as input. The library was multiplexed and sequenced using the Illumina HiSeq 2500 platform, yielding about 2 Gb of 150‐bp paired‐end reads for each sample. The plastid genome was assembled using GetOrganelle v.1.7.1 (Jin et al., 2020) with the default parameters. Each plastid genome was then annotated with PGA (Qu et al., 2019). We converted annotations into graphical maps using OGDRAW (Greiner et al., 2019). After format transfer using GB2sequin (Lehwark & Greiner, 2018), all plastome sequences were deposited in GenBank (accession nos., PQ154528–PQ154569; Table 1). In addition, nine plastomes of *Gentiana crassicaulis* were retrieved from GenBank for comparison analysis (Table 1).

### 2.3 Plastome structural changes and nucleotide diversity

Genome comparisons were conducted to identify intra‐species microstructural changes using mVISTA (Frazer et al., 2004). We analyzed genome rearrangement by using the progressive Mauve algorithm in Mauve v2.3.1 (Darling et al., 2010) using the plastid genome sequence with only one IR copy. The genes on the boundaries of the junction sites of the plastome were visualized in IRscope (Amiryousefi et al., 2018). To estimate nucleotide diversity, sequences of all genes, intergenic regions and RNA were extracted in PhyloSuite v.1.2.2 (Zhang, Gao, et al., 2020) and aligned using MAFFT v.7.313 (Katoh et al., 2002). Number of indel and nucleotide diversity (Pi) of different sequences were measured in DnaSP v.5 (Librado & Rozas, 2009). To test whether plastome diversity was correlated with plastome size, Mantel tests were performed in R to clarify the relationship between plastome size and Pi or number of indel.

### 2.4 Repeat sequence analysis

Microsatellites (SSRs) were identified by MISA (Beier et al., 2017) with the thresholds of 10, 6, 5, 5, 5, 5 repeated units for mono‐, di‐, tri‐, tetra‐, penta‐, and hexa‐nucleotide SSRs, respectively. We used Tandem Repeats Finder (Benson, 1999) to find the tandem repeated sequences with the default settings. We used REPuter (Kurtz et al., 2001) to identify the dispersed repeated sequences, including forward, reverse, complement, and palindromic repeats. The Hamming distance and minimum repeated size were set at three and 30 bp, respectively. To test whether repeats were correlated with plastome size, Mantel tests were performed in R to clarify the relationship between plastome size and number of SSRs, tandem repeated sequences and dispersed repeated sequences.

### 2.5 Phylogenetic analysis

To reconstruct intra‐species phylogenetic relationship in each of the target species, we built maximum likelihood (ML) trees using three matrices: WP included whole plastome after removing one IR, PCS contained all protein coding genes, IRS comprised intergenic regions. We examined each matrix and removed the most rapidly evolving sites using Gblocks v.0.91b (Talavera & Castresana, 2007) with default setting. The substitution model was chosen using ModelFinder v.2 (Kalyaanamoorthy et al., 2017). Phylogenetic analyses were inferred using IQ‐TREE v.1.6.8 (Nguyen et al., 2015) for 5000 ultrafast (Minh et al., 2013) bootstraps. The trees were visualized with R package ggtree (Yu et al., 2017).

## 3 RUSULTS

### 3.1 General plastome characteristics

**FIGURE 1:**
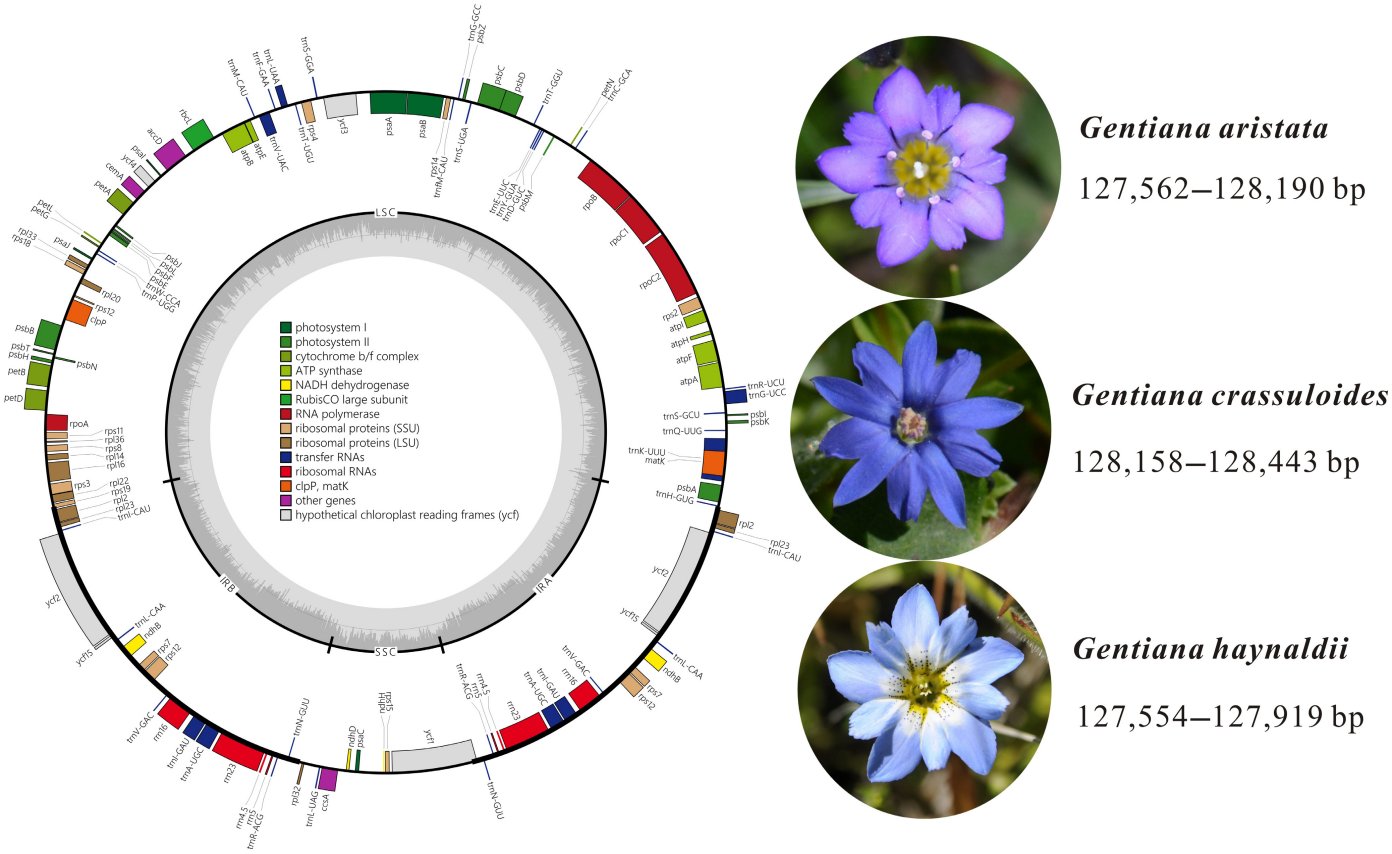
Schematic map of overall features of the chloroplast genome of three annual gentians in *Gentiana* section *Chondrophyllae* s.l. Genes drawn inside the circle are transcribed clockwise, and those drawn outside are transcribed counterclockwise. Genes belonging to different functional groups are shown in different colors.

**FIGURE 2:**
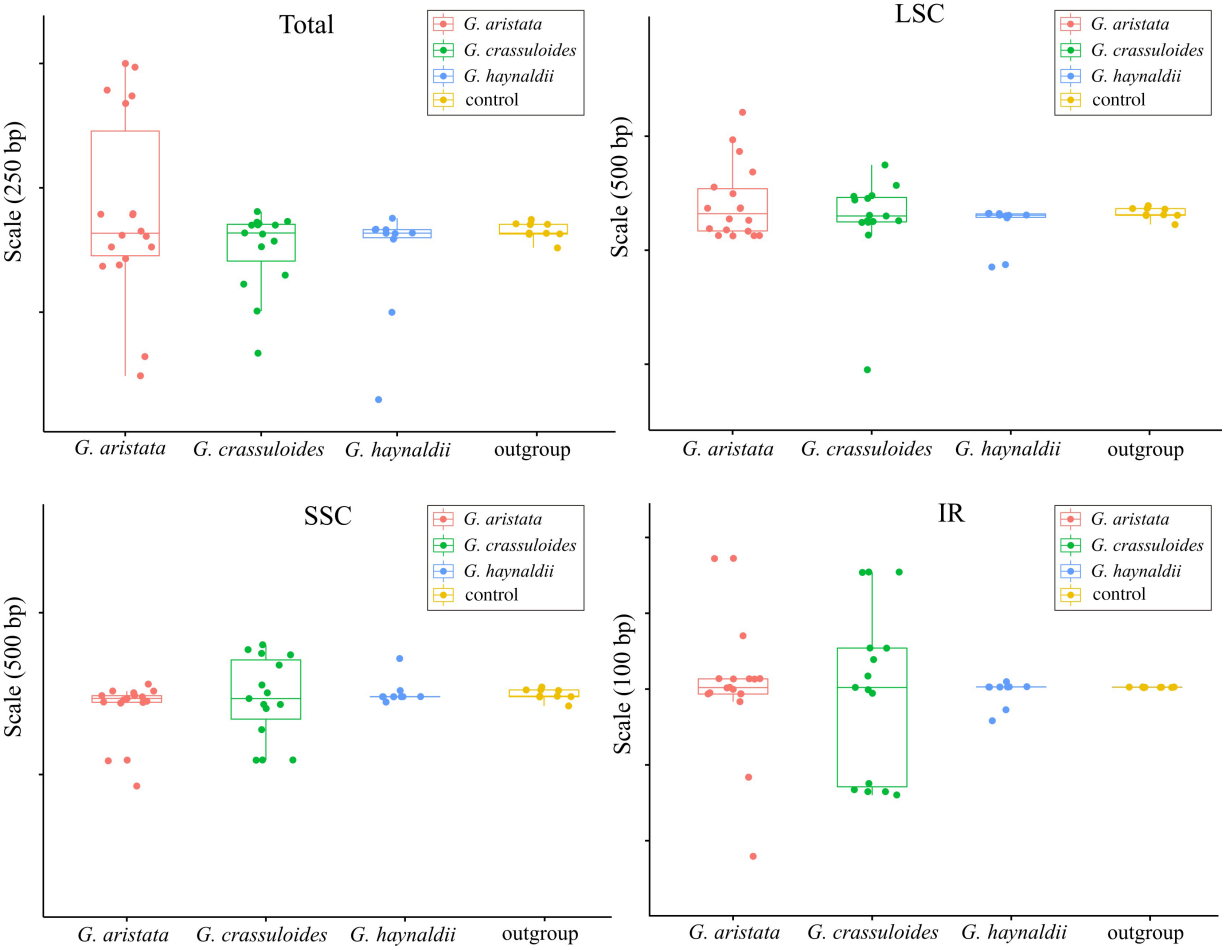
Plastome size variation in three annual gentians in *Gentiana* section *Chondrophyllae* s.l. The control is *Gentiana crassicaulis* that belongs to the sister group of section *Chondrophyllae* s.l. The median of each boxplot was aligned to keep in a line in each panel. The *y*‐axle shows the scale of size variation, and the scale for each interval is presented in bracket. IR, inverted repeat; LSC, long single copy; SSC, small single copy.

In this study, we newly sequenced and assembled 25 plastomes of *G. crassuloides* and *G. haynaldii*. The 15 plastomes of *G. crassuloides* varied from 128,158 to 128,443 bp in size, with variation of 285 bp (Figure 1). The size variation in LSC, IR and SSC is 898, 294 and 356 bp, respectively (Table 1; Figure 2). The 10 plastomes of *G. haynaldii* varied from 127,554 to 127,919 bp in size, with variation of 365 bp (Figure 1). The size variation in LSC, IR and SSC is 236, 52 and 135 bp, respectively (Table 1; Figure 2). The 18 plastomes of *G. aristata* assembled in Fu et al. (2024) were annotated in this study, and their size vary from 127,562 to 128,190 bp, with size variation of 628 bp (Figure 1). The size variation in LSC, IR and SSC is 542, 393 and 315 bp, respectively (Figure 2). All plastomes of the three species encoded a total of 105 unique genes, of which 18 were duplicated in IR regions. The 105 genes consist of 71 protein‐coding genes, 30 tRNA genes, and 4 rRNA genes. Regarding to the control species, based on 9 plastomes of *G. crassicaulis*, size variation in total plastome, LSC, IR and SSC was 64, 64, 1 and 1 bp, respectively (Figure 2).

### 3.2 Structural changes and nucleotide diversity

**FIGURE 3:**
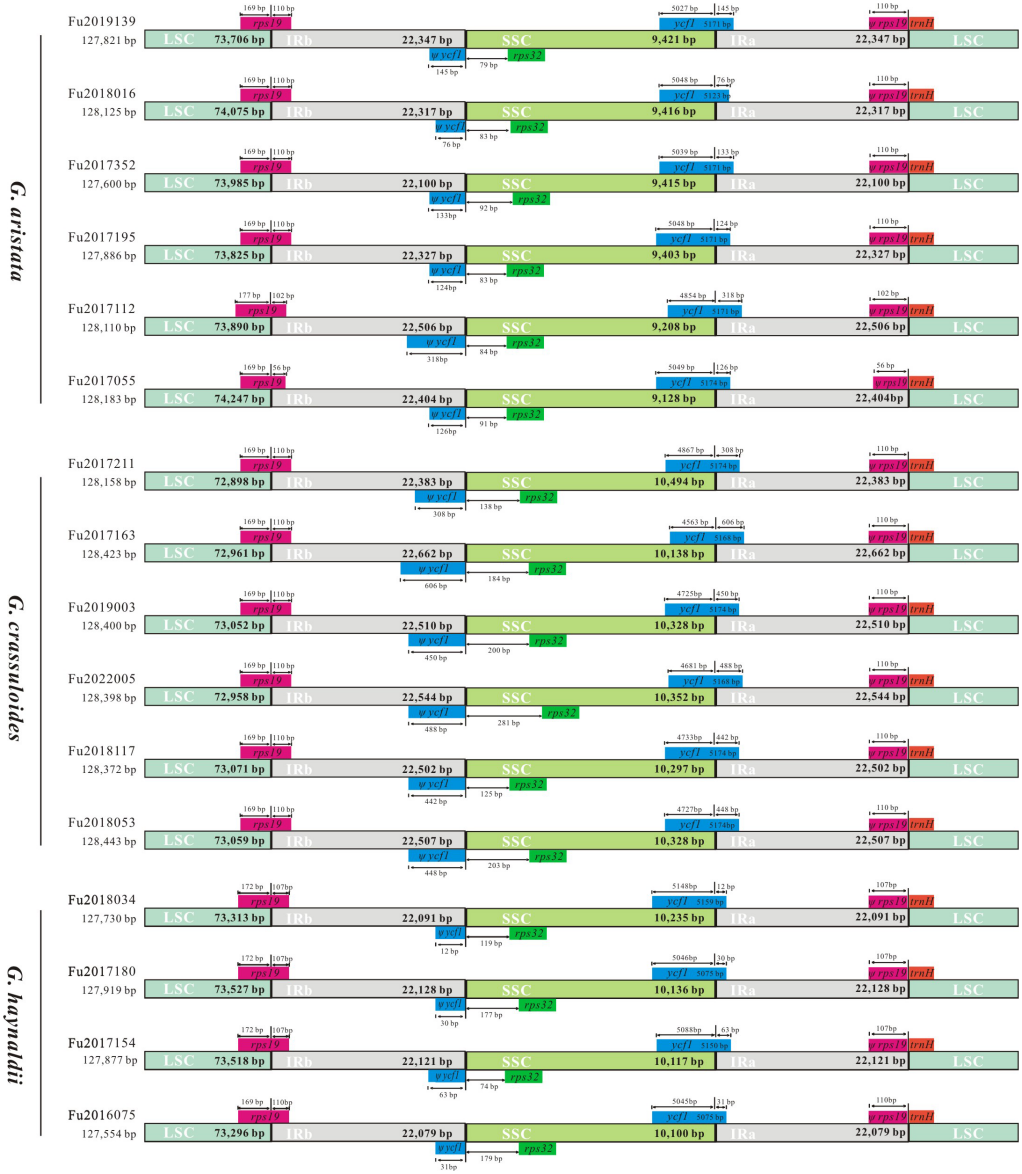
Comparison of LSC, IRs, and SSC junction positions among plastomes of three annual gentians in *Gentiana* section *Chondrophyllae* s.l.

Genome comparison showed nucleotide variation within each species, but did not detect either structural changes such as genome rearrangement (Figure S1) or gene loss (Figures S2–S4). We did not detect variation of gene composition in the boundaries of the junction sites in plastomes within each of the three gentians, but gene shift in the boundaries of the junction was observed (Figure 3). For example, the length of *rps19* and *ycf1* in the IR region was ranged from 56 to 110 bp, and 76 to 318 bp in *G. aristata*, respectively.

**FIGURE 4:**
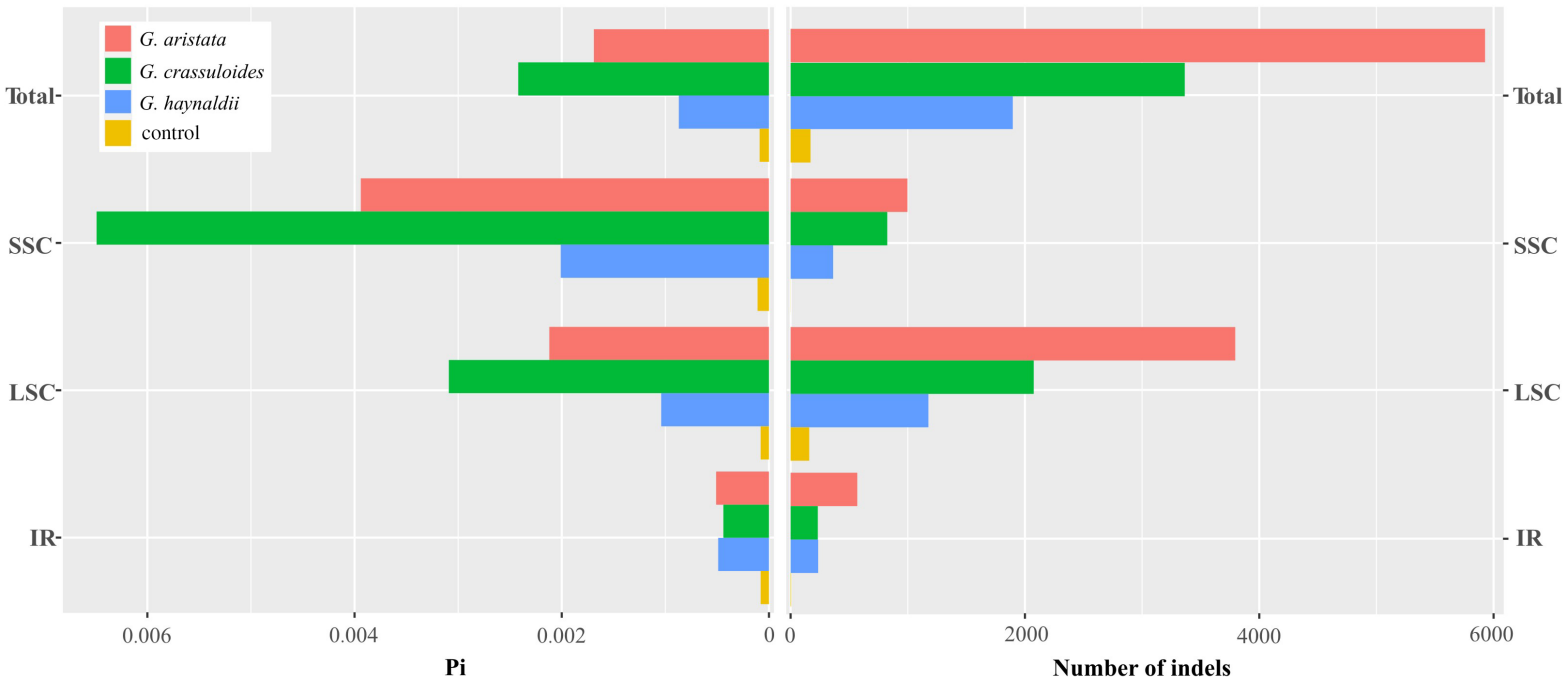
Nucleotide diversity (Pi) and number of indels in plastid genomes of three annual gentians in *Gentiana* section *Chondrophyllae* s.l. The results of total plastome, long single copy (LSC), small single copy (SSC) and inverted repeat (IR) are presented in turn. The control is *G. crassicaulis* that belongs to the sister group of section *Chondrophyllae* s.l.

In general, the target species had much higher nucleotide diversity than the control. Among the three target species, *G. crassicaulis* had the highest nucleotide diversity in the total plastome, LSC and SSC region, followed by *G. aristata* and then *G. haynaldii* (Table 2; Figure 4). In plastome, SSC region had highest nucleotide diversity in all the three species, and IR had the lowest. *G. aristata* had the largest number of indels in all regions of the plastome, and *G. haynaldii* had the lowest (Table 3; Figure 4). Mantel tests did not show significant correlation between plastome size and Pi or number of indels in LSC, SSC and total plastome (*p*‐value ranged from 0.1619 to 0.6272). The exception occurred in IR region, in which a significant relationship between Pi and IR size was detected (*p*‐value = 0.0065).

**TABLE 2:** Summary of intraspecific length variation in plastid genomes of four *Gentiana* species.

Species	No.	Total			LSC			IR			SSC		
		Min.	Max.	Variation	Min.	Max.	Variation	Min.	Max.	Variation	Min.	Max.	Variation
*G. aristata*	18	127,562	128,190	628	73,705	74,247	542	22,113	22,506	393	9128	9443	315
*G. crassuloides*	15	128,158	128,443	285	72,308	73,206	898	22,368	22,662	294	10,138	10,494	356
*G. haynaldii*	10	127,554	127,919	365	73,296	73,532	236	22,079	22,131	52	10,100	10,235	135
*G. crassicaulis*	9	148,724	148,788	64	81,110	81,174	64	25,271	25,272	1	17,070	17,071	1

*Note*: *Gentiana crassicaulis* was served as the control.

Abbreviations: IR, inverted repeat; LSC, large single‐copy; Max., maximum value; Min., minimum value; No., number of plastid genome; SSC, small single‐copy regions.

**FIGURE 5:**
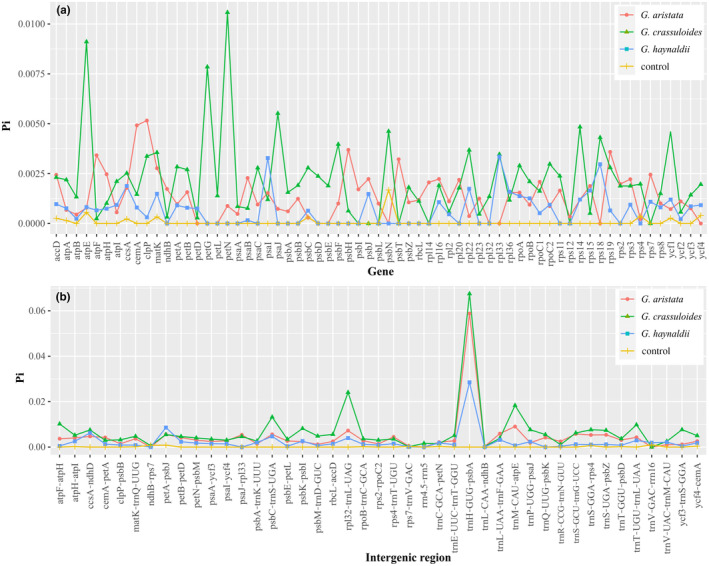
Nucleotide diversity (Pi) of protein coding genes (a) and intergenic regions (b) in plastid genomes of three gentians belonging to *Gentiana* section *Chondrophyllae* s.l. The control is *G. crassicaulis* that belongs to the sister group of section *Chondrophyllae* s.l.

Comparison of nucleotide diversity of protein coding genes indicated that *G. crassicaulis* had the highest average nucleotide diversity (Pi = 0.0023), followed by *G. aristata* (Pi = 0.0013) and then *G. haynaldii* (Pi = 0.0010). Nucleotide diversity varied among genes, for example, *atpE*, *petG* and *petN* had the highest value in *G. crassicaulis*, *cemA* and *clpP* had the highest value in *G. aristata*, and *psaI* had the highest value in *G. haynaldii* (Figure 5a). Regarding to intergenic regions, *G. crassicaulis* had the highest average nucleotide diversity (Pi = 0.0061), followed by *G. aristata* (Pi = 0.0040) and then *G. haynaldii* (Pi = 0.0023). Among tested intergenic regions, *trnH*‐*psbA* had the highest nucleotide diversity in all three target species (Figure 5b). *Rpl32*‐*trnL* and *psbC*‐*trnS* regions also had high nucleotide diversity within each of the three species. The control had much lower nucleotide diversity than the three target species in both genes and intergenic regions (Figure 5). Nucleotide diversity was very low in most RNAs in all tested species (Figure S5).

### 3.3 Repeat sequences

**FIGURE 6:**
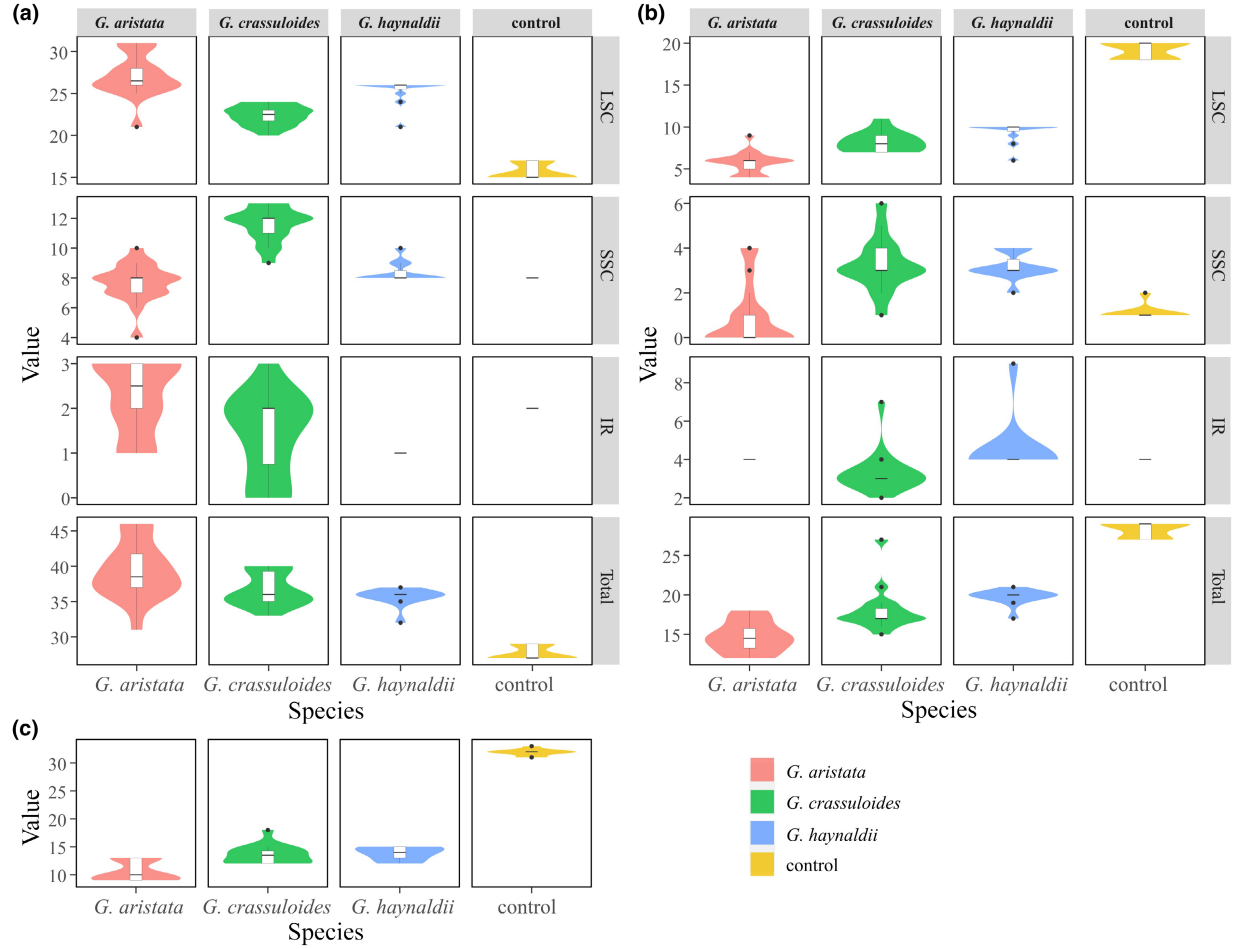
Results of repeat sequence in three annual gentians. (a) SSRs; (b) tandem repeated sequences; (c) dispersed repeated sequences. IR, inverted repeat; LSC, long single copy; SSC, small single copy.

The average number of detected SSRs in plastomes of *G. aristata*, *G. crassuloides*, *G. haynaldii* and control species was 39.3, 36.7, 35.6 and 27.7, respectively. In the three target species, most SSRs occurred in LSC (from 22.4 to 27.1), and rare in IR (from 1.0 to 2.3) (Figure 6a). As expected, the most detected SSR motif was mononucleotide. Regarding to tandem repeated sequences, the average number in *G. aristata*, *G. crassuloides*, *G. haynaldii* and control species was 14.7, 18.1, 19.7 and 28.2, respectively (Figure 6b). The average number of dispersed repeated sequences detected in plastomes of *G. aristata*, *G. crassuloides*, *G. haynaldii* and control species was 10.6, 13.6, 13.8 and 31.9, respectively (Figure 6c). Mantel tests showed a significant negative correlation between total plastome size and number of SSRs, and significant positive correlations between total plastome size and tandem repeated sequences and dispersed repeated sequences (*p*‐value ranged from 2.20e^−16^ to 1.02e^−11^). Among the three plastome regions, only size of LSC showed significant correlation with number of SSRs and tandem repeated sequences (*p*‐value was 2.27e^−10^ and 2.20e^−16^, respectively).

### 3.4 Phylogenetic analysis

**FIGURE 7:**
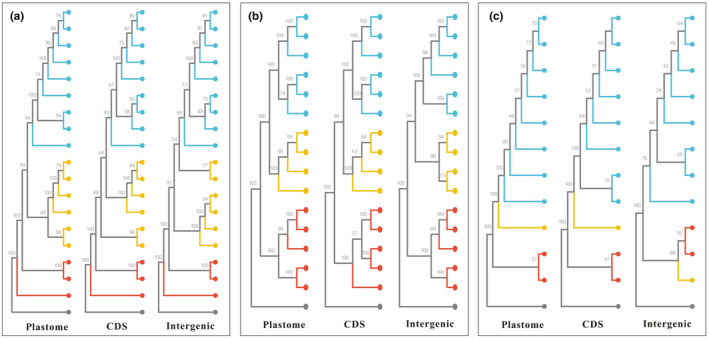
Phylogenetic relationship in *Gentiana aristata* (a), *G. crassuloides* (b) and *G. haynaldii* (c) based on three matrixes. The outgroup is presented with a gray dot in the base of each tree.

The three matrix in each species resulted in various backbone topologies in the ML trees in which most of deep nodes were highly supported (Figure 7). In *G. aristata*, the yellow clade being a monophyletic group was weakly supported (bootstrap supporting, BS = 69%) by the WP matrix, but was a polyphyletic group based on PCS and IRS matrixes (Figure 7a). In *G. crassuloides*, the three matrixes produced the same backbone topology and very similar BS values in deep nodes (Figure 7b). Regarding to *G. haynaldii*, besides the poor clades existed within the species, the position of the yellow clade was identical based on the WP and PCS matrixes, but differed with the result based on the IRS matrix (Figure 7c).

## 4 DISCUSSION

By sampling and sequencing individuals covering the range of three species belonging to various series in *Gentiana* section *Chondrophyllae* s.l., our results indicated high intra‐species plastome diversity in the speciose lineage. The three target species, *G. aristata*, *G. crassuloides* and *G. haynaldii*, had much higher plastome diversity than the control species belonging to the sister group of section *Chondrophyllae* s.l. For example, the three species had much higher values in plastome size variation, Pi and number of indels than the control species (Figures 2 and 4, 5, 6). The high level of plastome diversity is mainly caused by two reasons. First, large plastome size variation due to a large number of indels covering the whole plastome (Figure 4), rather than structural changes such as expansion/contraction of SSC or IR which commonly detected among species in this section (Fu et al., 2021, 2022). The indels detected in the three target species were much higher (from 1896 to 5927) than in species from *G*. sect. *Monopodiae* (e.g., 441 or 581 in plastome; Mao et al., 2023). Second, high nucleotide polymorphism in both genes and intergenic regions. The high nucleotide polymorphism in *Chondrophyllae* species is much higher than the control species in *G*. section *Cruciata* (Figure 5) and two species in *G*. sect. *Monopodiae* whose average Pi in genes and intergenic regions were lower than 0.001 and 0.0025, respectively (Mao et al., 2023). Within the three target species, *G. crassuloides* had the highest plastome diversity, for example large size variation and Pi in SSC and IR, and high Pi in genes and intergenic regions. In particular, as most conserved region in plastome (Guisinger et al., 2011), IR showed the largest size variation in *G. crassuloides*. The high plastome diversity which could afford more informative sites shall be one of the reasons why the three matrix of *G. crassuloides* resulted in the same backbone topology in phylogenetic analysis (Figure 7b), although higher diversity did not always mean the better phylogenetic supports due to diverse factors such as the different evolutionary history of genes. In addition, the three annual gentians with high nucleotide diversity had high level of SSRs and repeats, consistent with the observation in Malvaceae that correlations occurred among repeats, SSRs and indels (Abdullah et al., 2021).

**TABLE 3:** Summary of intraspecific nucleotide diversity in plastid genomes of four *Gentiana* species.

Species	Region	No. PS	No. indel	Pi	k
*G. aristata*	LSC	971	3795	0.00212	153.731
	IR	67	569	0.00051	11.164
	SSC	229	997	0.00394	35.474
	Total	1334	5927	0.00169	211.532
*G. crassuloides*	LSC	774	2075	0.00309	223.342
	IR	40	232	0.00044	9.933
	SSC	219	825	0.00649	65.067
	Total	1070	3364	0.00242	307.300
*G. haynaldii*	LSC	314	1176	0.00104	75.691
	IR	35	235	0.00049	10.727
	SSC	81	363	0.00201	19.927
	Total	443	1896	0.00087	110.018
*G. crassicaulis*	LSC	27	159	0.00008	6.889
	IR	9	5	0.00008	2.000
	SSC	7	1	0.00011	1.889
	Total	52	170	0.00009	12.778

Abbreviations: k, average number of nucleotide differences; No. indel, number of indels; No. PS, number of polymorphic sites; Pi., nucleotide diversity.

Previous study indicated that repeats could yield variable‐length insertions and deletions, and thereby repeat‐mediated genome rearrangement was linked with plastid genome variability (Gurdon & Maliga, 2014). Comparing to the control species, much larger number of SSRs and smaller number of tandem repeated sequences and dispersed repeated sequences were detected in the three *Chondrophyllae* species (Figure 6). The number of SSRs was variable within species in sect. *Chondrophyllae* s. l., and most SSRs were mono‐nucleotide repeats, being consistent with other gentians (Mao et al., 2023) and angiosperms (e.g., Li et al., 2023; Mwanzia et al., 2020; Xu et al., 2022). We found that repeat content was significantly correlated with genome size, as plastid with smaller genome size had more SSRs and less repeat elements (dispersed repeat and tandem repeat) in *Gentiana*. Previous studies also detected that repeat content was positively correlated with genome size and genomic rearrangements, for example in *Medicago* (Wu et al., 2021) and Alismatidae (Li et al., 2023). Since the repeat elements and plastome size are significantly correlated, we speculate that repeat elements are another factor likely contribute to plastome size variation in *G*. sect. *Chondrophyllae* s. l., being consistent with the case in Alismatidae (Li et al., 2023). Variation in repeat elements and plastome degradation such as *ndh* complex loss (Fu et al., 2022) may be the key factors explaining the genome size variation in sect. *Chondrophyllae* s. l. In addition, accelerated plastid genome evolution may contribute to the early stages of the speciation process by increasing the likelihood of intraspecific cyto‐nuclear genetic incompatibilities (Barnard‐Kubow et al., 2014). We detected much higher intraspecific diversity in species of sect. *Chondrophyllae* s. l. than its sister group, hinting accelerated plastid genome evolution. In fact, elevated substitution rate in plastome genes was indeed observed in sect. *Chondrophyllae* s. l. under broader context (Fu et al., 2021). Therefore, rapid plastid genome evolution maybe one reason explaining the high species diversity in sect. *Chondrophyllae* s. l.

Plastome was widely used to analyze phylogenetic relationship in both low and high taxonomic units, and could provide robust results in most cases (Lv et al., 2023; Zhou et al., 2022, 2023). Our results based on three matrices showed conflicting phylogenetic signals in two of three species (Figure 7a,c). In fact, conflicting phylogenetic signals in the plastome were observed in various lineages (Walker et al., 2019), from genus level such as *Rhododendron* (Mo et al., 2022) to higher level such as Leguminosae (Zhang, Wang, et al., 2020), Laureae (Xiao et al., 2020) and Fagales (Yang et al., 2021). It is still uncertain why the conflict in plastome‐inferred phylogenies occurred, and suggested potential reason including heteroplasmic recombination in plastome (Mo et al., 2022; Walker et al., 2019) and complex history of plastome structural evolution (Zhang, Wang, et al., 2020). Currently, no available study detects occurrence of plastid recombination in sect. *Chondrophyllae* s. l., but heteroplasmy was clarified in an annual gentian in sect. *Microsperma* T.N. Ho (Sun et al., 2019), suggesting that plastid recombination could not be ruled out in *Chondrophyllae* species. In addition, hybridization, which could lead to plastid recombination, was proved to be common in sect. *Chondrophyllae* s. l. (Chen et al., 2021; Fu et al., 2022), including rampant hybridization within *G. aristata* (Fu et al., 2024). We thereby suggested that heteroplasmic recombination in plastome of sect. *Chondrophyllae* s. l. shall be possible, but direct evidences are needed. We also found that the species having the highest diversity had consistent phylogenetic topology among the three datasets (Figure 7b) and indicate that conflict in phylogenetic backbone may be easier to be detected in taxon with less plastome sequence diversity. Therefore, heteroplasmic recombination and poor sequence diversity maybe the potential reasons of the conflict in phylogenetic signals, but it is still early to draw a firm conclusion.

## AUTHOR CONTRIBUTIONS

**Shan‐Shan Sun:** Data curation (equal); funding acquisition (equal); project administration (equal); writing – original draft (equal). **Zhi‐Yong Pan:** Formal analysis (equal); visualization (equal). **Yu Fu:** Formal analysis (equal); visualization (equal). **Shen‐Jue Wang:** Formal analysis (equal); visualization (equal). **Peng‐Cheng Fu:** Formal analysis (equal); investigation (equal); writing – review and editing (equal).

## CONFLICT OF INTEREST STATEMENT

None declared.

## Supporting information


Figures S1–S5.

## Data Availability

All data are provided within the text, tables, figures and supplements. The raw plastome sequences and annotations are provided in FigShare with the link of https://doi.org/10.6084/m9.figshare.25479385.v1.
