# Supplementary material for: Rampant intraspecific variation of plastid genomes in Gentiana section Chondrophyllae
Source: Ecol Evol. 2024 Sep 2;14(9):e70239. doi: 10.1002/ece3.70239 (PMC11368500; doi:10.1002/ece3.70239)
Supplement: Supplementary file 1 — Figures S1–S5. [file ECE3-14-e70239-s001.docx]

**
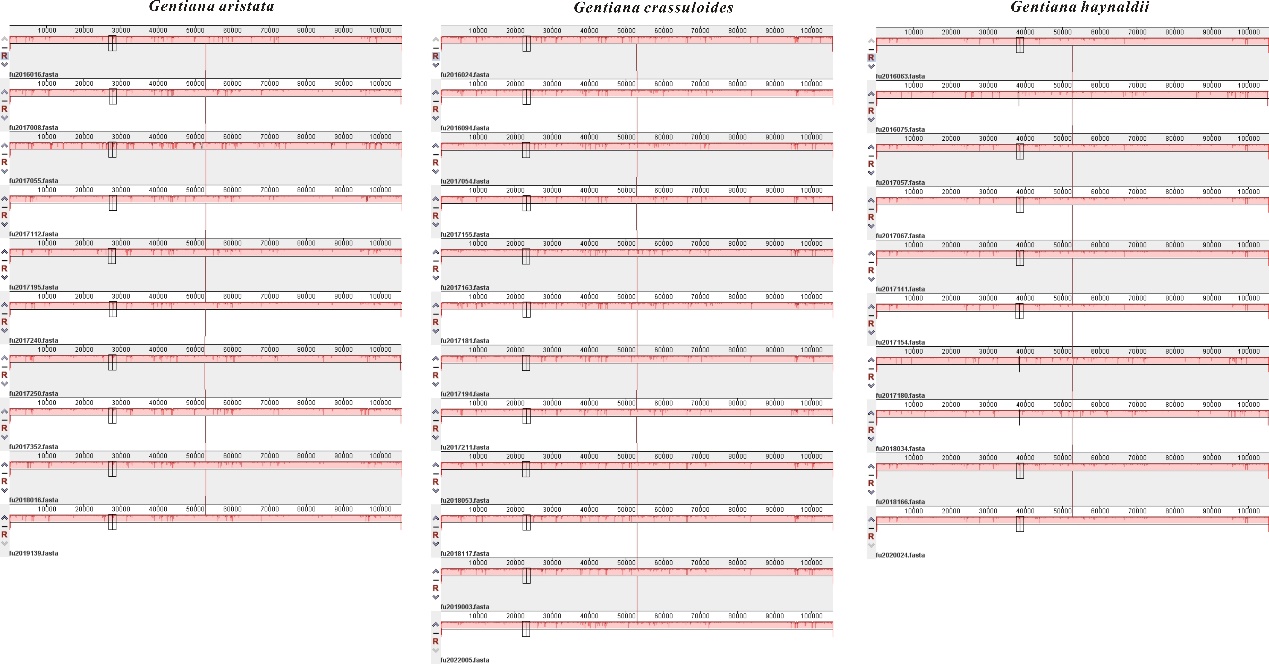
**

**Figure S1** Results of plastome alignment in the three gentians.

**
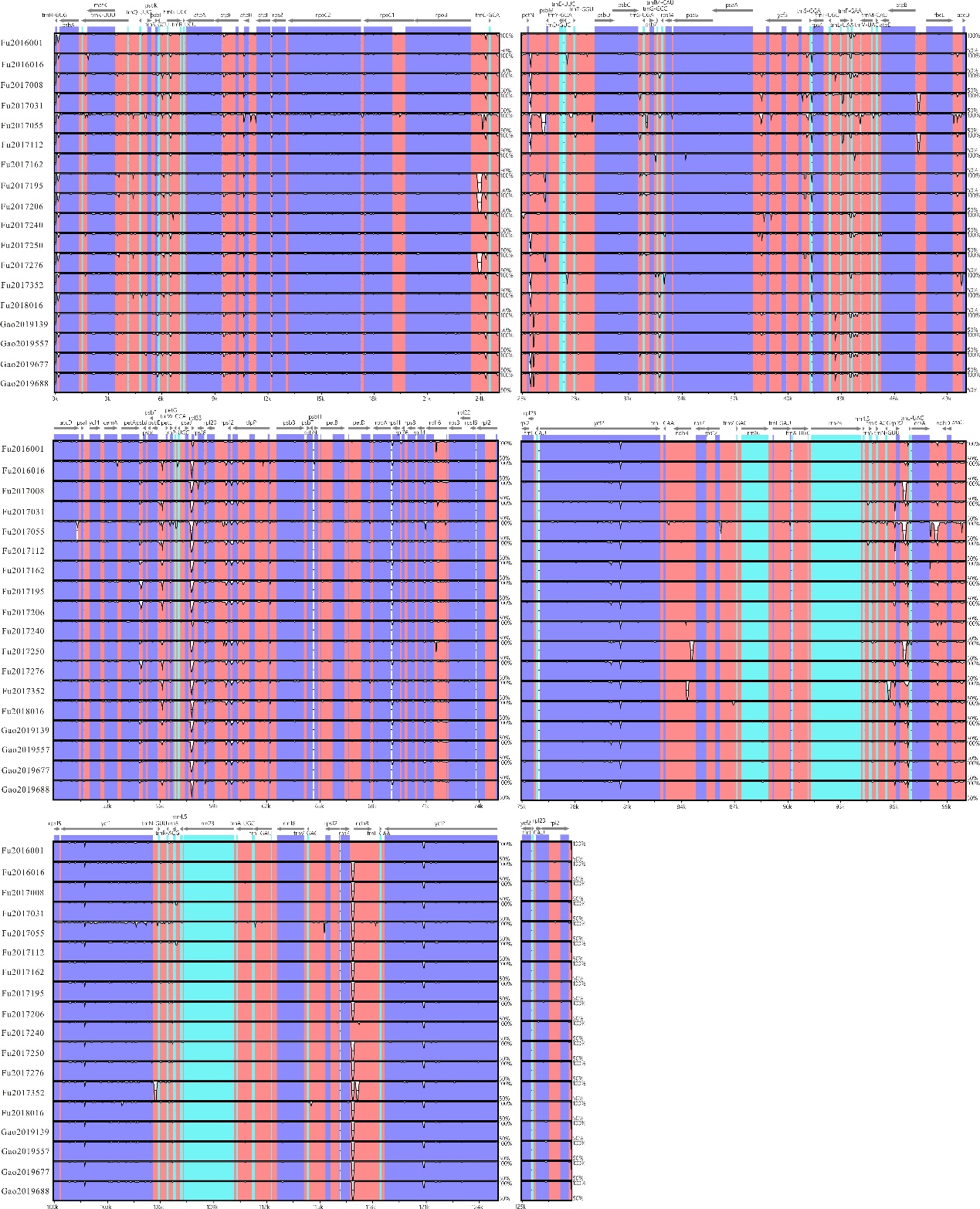
Figure S2** Plastome comparison among *Gentiana* *aristata* individuals using *G. straminea* as the reference. The arrows on the top show the direction of genes. The colors blue, light blue, red and white represent genes, rRNAs, intergenic regions and sequence losses.

**
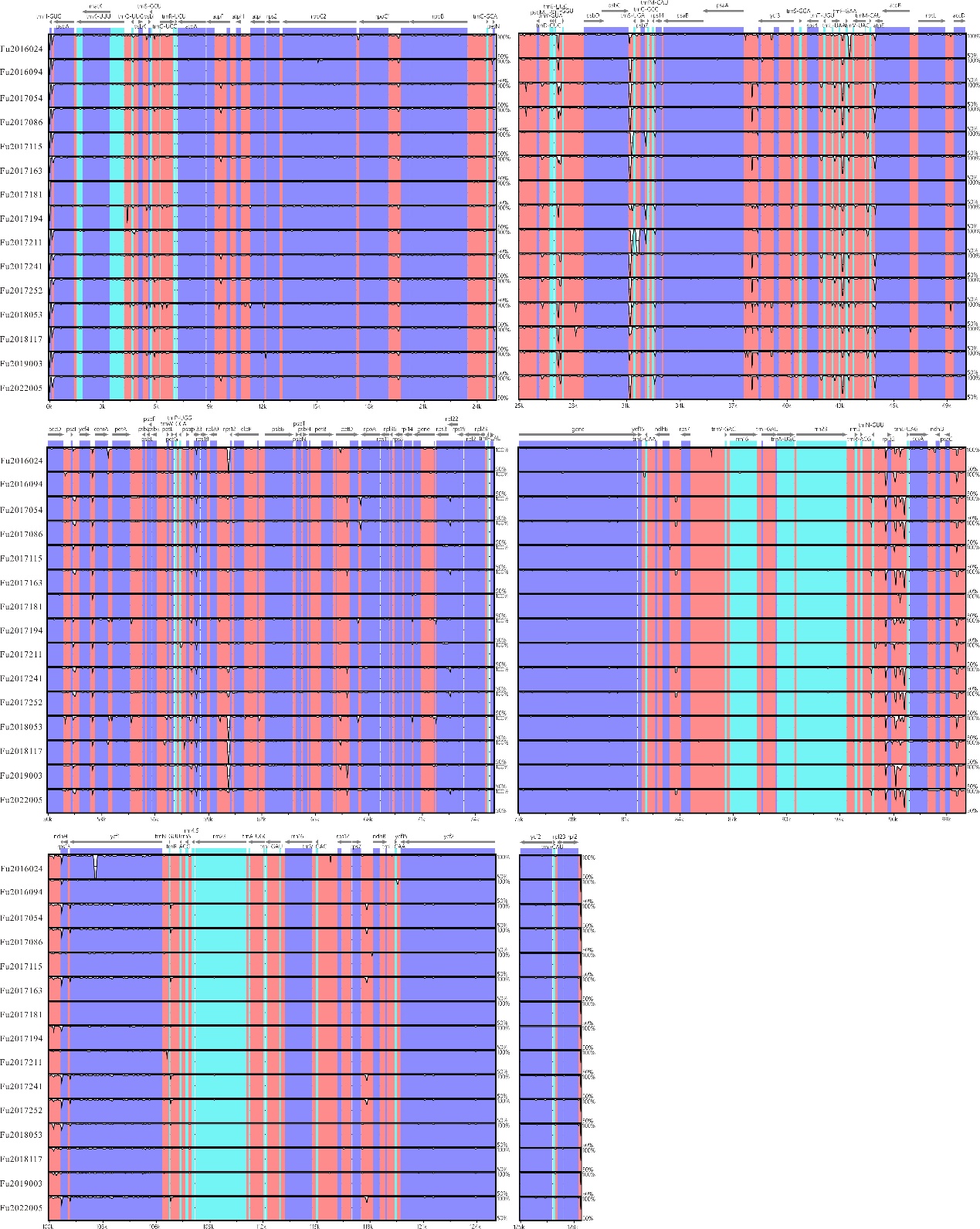
Figure S3** Plastome comparison among *Gentiana* *crassuloides* individuals using *G. straminea* as the reference. The arrows on the top show the direction of genes. The colors blue, light blue, red and white represent genes, rRNAs, intergenic regions and sequence losses.

**
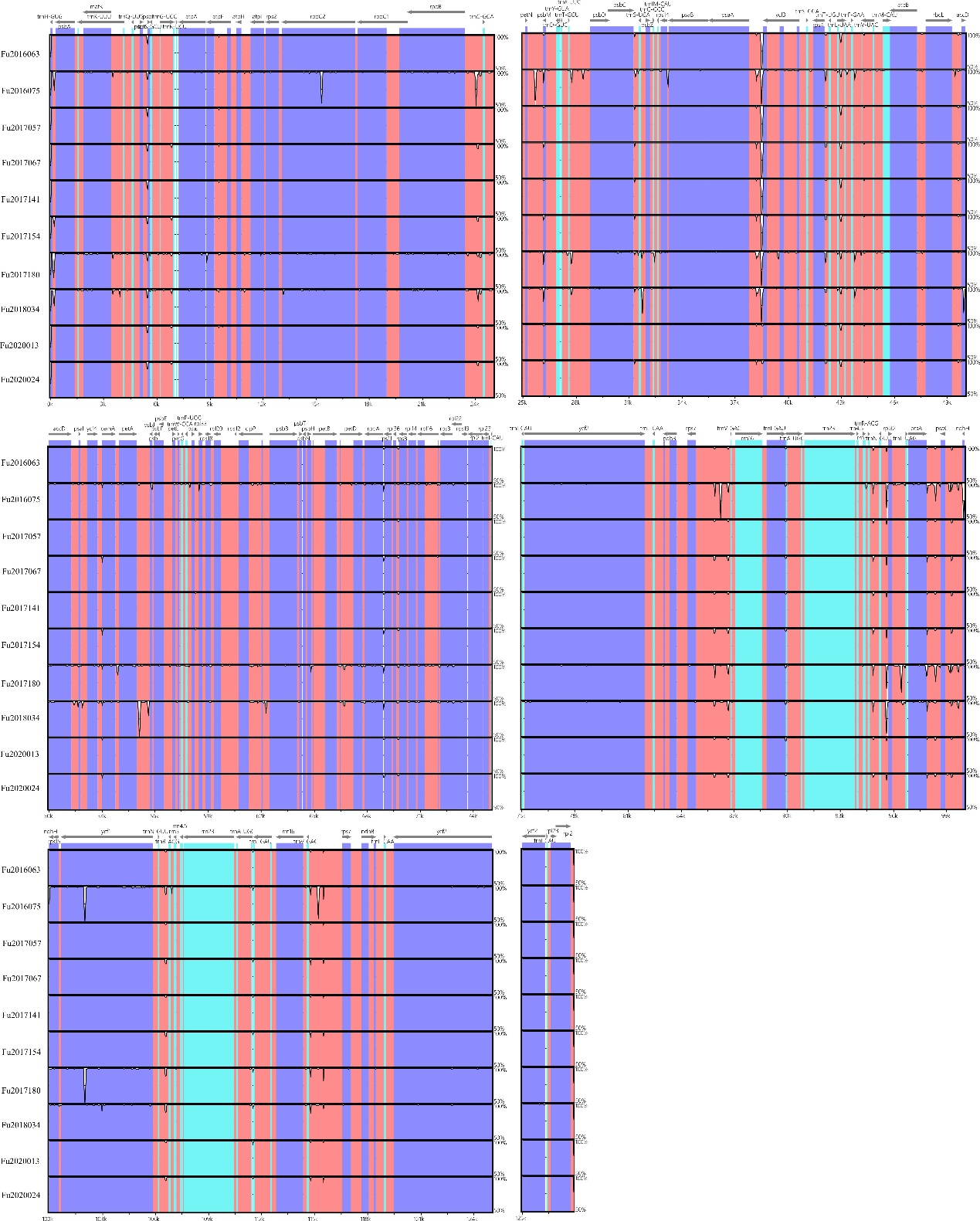
Figure S4** Plastome comparison among *Gentiana* *haynaldii* individuals using *G. straminea* as the reference. The arrows on the top show the direction of genes. The colors blue, light blue, red and white represent genes, rRNAs, intergenic regions and sequence losses.

**
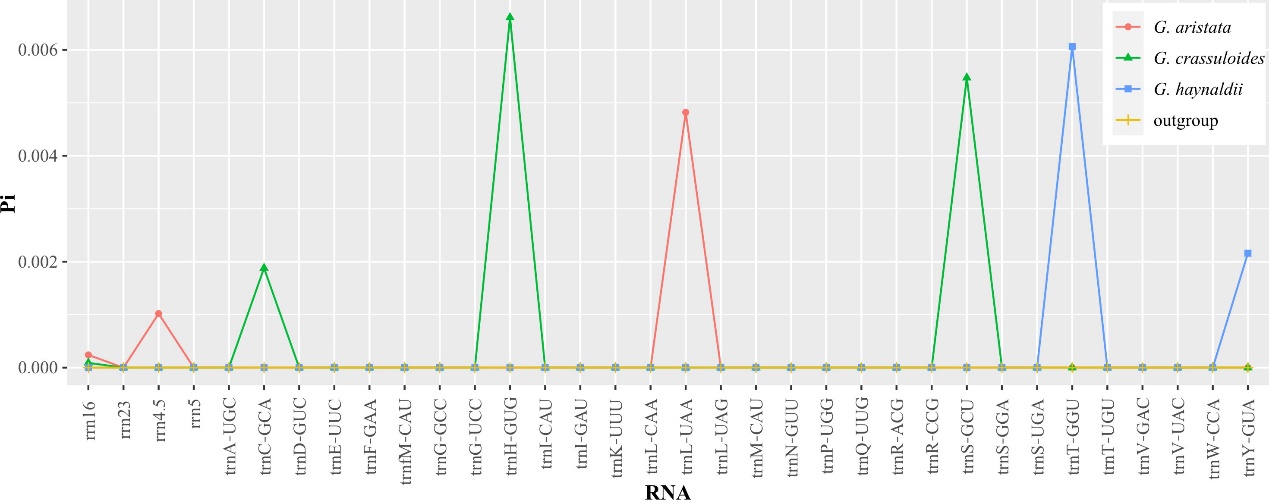
Figure S5** Nucleotide diversity (Pi) of RNA in plastid genomes of three gentians belonging to *Gentiana* section *Chondrophyllae* s.l. The control is *G. crassicaulis* that belongs to the sister group of section *Chondrophyllae* s.l.
